# Plasma albumin levels predict risk for nonsurvivors in critically ill patients with COVID-19

**DOI:** 10.2217/bmm-2020-0254

**Published:** 2020-06-03

**Authors:** Juyi Li, Meng Li, Shasha Zheng, Menglan Li, Minghua Zhang, Minxian Sun, Xiang Li, Aiping Deng, Yi Cai, Hongmei Zhang

**Affiliations:** ^1^Department of Pharmacy, Key Laboratory for Molecular Diagnosis of Hubei Province, The Central Hospital of Wuhan, Tongji Medical College, Huazhong University of Science & Technology, Wuhan, Hubei 430021, China; ^2^Department of Endocrinology, The Central Hospital of Wuhan, Tongji Medical College, Huazhong University of Science & Technology, Wuhan, Hubei 430021, China; ^3^Department of Pulmonary & Critical Care Medicine, The Central Hospital of Wuhan, Tongji Medical College, Huazhong University of Science & Technology, Wuhan, Hubei 430021, China; ^4^Department of Radiology, The Central Hospital of Wuhan, Tongji Medical College, Huazhong University of Science & Technology, Wuhan, Hubei 430021, China; ^5^Department of Pain, The Central Hospital of Wuhan, Tongji Medical College, Huazhong University of Science & Technology, Wuhan, Hubei 430021, China

**Keywords:** albumin, biomarkers, COVID-19, critically ill patients, infection, pneumonia

## Abstract

**Aim:** We aimed to explore the biomarkers for disease progression or the risk of nonsurvivors. **Materials & methods:** This study included 134 hospitalized patients with confirmed COVID-19 infection. The outcome of moderate versus severe versus critically ill patients and survivors versus nonsurvivors were compared. **Results:** An increase in the severity of COVID-19 pneumonia was positively associated with lower levels of platelets and albumin (all p < 0.05). In the critical group, the plasma levels of albumin continued to have a significant association for the risk of nonsurvivors (p < 0.05), even after adjusting for confounding factors. **Conclusion:** Albumin levels could be used as an independent predictor of the risk of nonsurvivors in critically ill patients with COVID-19.

A series of unexplained pneumonia cases (with a history of work or residence around the Huanan seafood wholesale market) were admitted to a hospital in Wuhan, Hubei province, China. Their clinical presentations were similar to viral pneumonia and some patients rapidly developed life-threatening acute respiratory diseases (ARDS) [1]. A novel coronavirus was then identified by sequencing the whole genome of the virus isolated from the patients and was named COVID-19 by the WHO [2,3]. To date, more than 80,000 confirmed cases have been identified in 34 provinces of China, more than 49,000 are from Wuhan city and the virus has been found in Japan, Thailand, South Korea, USA, etc [4,5].

Generally, the majority of COVID-19-positive patients are present with general symptoms of respiratory infection with a case fatality rate of 1.4–4% [3,6,7]. In some cases that develop severe or critical illness, death may be due to massive alveolar damage and progressive respiratory failure, with a higher mortality rate (38–60%) [8,9]. However, little is known regarding the clinical markers for the risk of nonsurvivors in patients with COVID-19.

The purpose of this study was to explore biomarkers for disease progression and the risk of nonsurvivors. We hope that our research will help clinicians identify patients with a high risk of nonsurvivors at an early stage.

## Materials & methods

### Study design & participants

In this retrospective study, we included discharged patients, including deaths, hospitalized with COVID-19 pneumonia in the Central Hospital of Wuhan from 1 January to 20 February 2020. COVID-19 was defined as a positive result on real time reverse transcriptase PCR and ‘ground-glass opacity’ on computed tomography (CT). This study was approved by the Ethics Commission of the Central Hospital of Wuhan. Written informed consent was waived by the Ethics Commission of the designated hospital under the criteria of emerging infectious diseases. The classification of diseases used is as described previously [10,11].

### Participants’ characteristics & data collection

This study retrospectively analyzed the patients’ medical history, epidemiological data (including workplace), history of disease exposure, fever, cough, headache, diarrhea and chest pain, etc. The laboratory tests included liver function, kidney function, blood cell count, COVID-19 nucleic acid and tests for other respiratory viruses etc. Data regarding medical expenses, lung CT image, drugs prescribed and comorbidities were also analyzed [12].

### Clinical outcomes

This study focused on discharged patients. The two patient subtypes included rehabilitation discharges and death cases.

### Statistical analysis

All data were expressed as median interquartile range (IQR), or percentages (%). Categorical data were tested using Fisher’s exact test or *x*^2^ test. Normal distribution data were tested by independent *t*-test, while non-normal distribution data were tested by nonparametric Mann–Whitney *U* test. A binary logistic regression analysis was used to assess the independent predictors for the risk of nonsurvivors. To predict the risk of nonsurvivors, a receiver operating characteristic curve was plotted to determine the cut-off point for albumin. The data were analyzed using SPSS 20.0. A two-sided α score <0.05 was considered statistically significant.

## Results

In this retrospective study, we included 134 discharged patients, including deaths. Patient demographics, characteristics, outcomes and medical expenses are summarized in Table 1. The median age of all the patients was 61.00 years, 69 (51.49%) of the patients were >60 years of age, 65 (48.51%) were <60 years of age and 75 (55.97%) of them were males. A total of 83.58, 96.27 and 100.00% had no history of smoking, drinking or a history of exposure to the Huanan seafood market, respectively. A total of 15 (11.19%) of the patients with COVID-19 were medical staff. Some patients had comorbidities including cardiovascular disease (44.03%), endocrine disorder (diabetes) (25.37%), digestive disorder (14.93%), respiratory disease (8.21%), neurological disease (17.16%) and solid tumor (9.70%). The median hospital stays and medical expenses for all the patients were 13.00 days and 24,093.38 yuan, respectively. Forty two (31.34%) patients died due to COVID-19 pneumonia.

**Table 1.** Demographics, characteristics, outcomes and medical expenses of patients with COVID-19.

Characteristics	Patients				p-value
	All (n = 134)	Moderate (n = 45)	Severe (n = 30)	Critical (n = 59)	
**Age, median (IQR), years**	61.00 (46.75–69.25)	50.00 (31.00–63.00)	59.50 (52.75–67.75)	67.00 (56.00–75.00)	0.000
<60 (%)	65 (48.51)	31 (68.89)	15 (50.00)	19 (32.20)	0.001
≧60 (%)	69 (51.49)	14 (31.11)	15 (50.00)	40 (67.80)	
**Gender, (%)**					
Females	59 (44.03)	21 (46.67)	15 (50.00)	23 (38.98)	0.557
Males	75 (55.97)	24 (53.33)	15 (50.00)	36 (61.02)	
**Smoking, (%)**					
Yes	22 (16.42)	8 (17.78)	5 (16.67)	9 (15.25)	0.942
No	112 (83.58)	37 (82.22)	25 (83.33)	50 (84.75)	
**Drinking, (%)**					
Yes	5 (3.73)	3 (6.67)	1 (3.33)	1 (1.69)	0.412
No	129 (96.27)	42 (93.33)	29 (96.67)	58 (98.31)	
**Exposure to Huanan seafood market, (%)**					
Yes	0 (0)	0 (0)	0 (0)	0 (0)	NA
No	134 (100)	45 (100.00)	30 (100.00)	59 (100.00)	
**Occupation, (%)**					
Medical staff	15 (11.19)	8 (17.78)	5 (3.73)	2 (3.39)	0.039
Nonmedical staff	119 (88.81)	37 (82.22)	25 (83.33)	57 (96.61)	
**Chronic disease, (%)**					
Cardiovascular disease	59 (44.03)	12 (40.00)	14 (46.67)	33 (55.93)	0.014
Hypertension	44 (32.84)	10 (33.33)	11 (36.67)	23 (38.98)	0.173
Endocrine disorder (diabetes)	34 (25.37)	6 (20.00)	10 (33.33)	18 (30.51)	0.072
Digestive disorder	20 (14.93)	5 (11.11)	3 (9.99)	12 (20.34)	0.294
Respiratory disease	11 (8.21)	2 (4.44)	1 (3.33)	8 (13.56)	0.133
Neurological disease	23 (17.16)	7 (15.56)	5 (3.73)	11 (18.64)	0.915
Solid tumor	13 (9.70)	5 (11.11)	3 (9.99)	5 (8.47)	0.902
**Hospital stays, median (IQR), days**	13.00 (10.00–20.00)	14.00 (11.00–20.00)	15.50 (12.75–22.00)	11.00 (7.00–17.00)	0.009
**Clinical outcomes, (%)**					
Rehabilitation discharge	92 (68.66)	45 (100.00)	30 (100.00)	17 (28.81)	0.000
Died	42 (31.34)	0 (0)	0 (0)	42 (71.19)	
**Medical expenses, median (IQR), yuan (RMB)**	24,093.38 (10,217.39–44,511.04)	13,588.28 (8825.39–30,426.43)	24,169.73 (11,221.73–34,855.10)	36,895.44 (12,726.83–61,336.93)	0.000

According to the severity of COVID-19 pneumonia (Table 1), the increase in the median age, percentage of patients ≥60 years, percentage of patients with cardiovascular disease, and the median medical expenses occurred concomitantly with the severity of COVID-19 pneumonia (all p-values <0.05). However, the median hospital stay was the shortest in the critical group, as this may have been due to the quick disease progression and early patient death.

The clinical characteristics of the patients are summarized in Table 2. The most common symptoms were fever (84.33%), cough (76.87%), diarrhea (20.15%), muscle pain (19.40%), generally feeling sick and vomiting (11.19%), as well as 96.27% experiencing more than one symptom. Out of the 134 patients, 62 (46.27%) developed ARDS and 120 (89.55%) had bilateral pneumonia. Most were treated with oxygen (66.42%), antibiotics (97.76%), antiviral drugs (97.76%), hormones (66.42%) and immunoglobulin (59.70%). An increase in the percentage of patients with bilateral pneumonia and hormones used occurred concomitantly with an increase in the severity of COVID-19 pneumonia (all p < 0.05).

**Table 2.** Clinical characteristics and treatment strategies of patients with COVID-19.

Characteristics	Patients				p-value
	All (n = 134)	Moderate (n = 45)	Severe (n = 30)	Critical (n = 59)	
**Clinical symptoms, (%)**					
Fever	113 (84.33)	36 (80.00)	25 (83.33)	52 (88.14)	0.520
Cough	103 (76.87)	32 (71.11)	21 (70.00)	50 (84.75)	0.158
Stuffy nose	2 (1.49)	0 (0)	1 (3.33)	1 (1.69)	0.499
Runny nose	2 (1.49)	0 (0)	1 (3.33)	1 (1.69)	0.499
Sneeze	0 (0)	0 (0)	0 (0)	0 (0)	NA
Sore throat	5 (3.73)	0 (0)	1 (3.33)	4 (6.78)	0.194
Chest pain	9 (6.72)	5 (11.11)	1 (3.33)	3 (5.08)	0.335
Diarrhea	27 (20.15)	6 (13.33)	10 (33.33)	11 (18.64)	0.099
Headache	8 (5.97)	3 (6.67)	1 (3.33)	4 (6.78)	0.787
Muscle pain	26 (19.40)	6 (13.33)	8 (26.67)	12 (20.34)	0.349
Feel sick and vomit	15 (11.19)	8 (17.78)	2 (6.66)	5 (8.47)	0.221
More than one symptom	129 (96.27)	41 (91.11)	29 (96.67)	59 (100.00)	0.060
**Complications, (%)**					
Acute kidney injury	5 (3.73)	0 (0)	0 (0)	5 (8.47)	0.037
Acute respiratory injury	4 (2.99)	0 (0)	0 (0)	4 (6.78)	0.073
Septic shock	2 (1.49)	0 (0)	0 (0)	2 (3.39)	0.275
Ventilator-associated pneumonia	1 (0.75)	0 (0)	0 (0)	1 (1.69)	0.527
Acute respiratory distress syndrome	56 (41.79)	0 (0)	5 (16.67)	51 (86.44)	0.000
**Lung CT images, (%)**					
Unilateral pneumonia	13 (9.70)	10 (22.22)	2 (6.66)	1 (1.69)	0.002
Bilateral pneumonia	120 (89.55)	35 (77.78)	27 (90.00)	58 (98.31)	0.003
Ground-glass turbidity	117 (87.31)	41 (91.11)	27 (90.00)	49 (83.05)	0.417
**Treatment strategies, (%)**					
Oxygen therapy	89 (66.42)	0 (0)	30 (100.00)	59 (100.00)	0.000
Extracorporeal membrane oxygenation	1 (0.75)	0 (0)	0 (0)	1 (1.69)	0.527
Antibiotics	131 (97.76)	44 (97.78)	30 (100.00)	57 (96.61)	0.593
Antiviral drugs	131 (97.76)	45 (100.00)	30 (100.00)	56 (94.92)	0.142
Antifungal drugs	5 (3.73)	0 (0)	1 (3.33)	4 (6.78)	0.194
Hormones	89 (66.42)	23 (51.11)	18 (60.00)	48 (81.36)	0.004
Immunoglobulin	80 (59.70)	25 (55.55)	19 (63.33)	36 (61.02)	0.768
Kidney replacement therapy	1 (0.75)	0 (0)	0 (0)	1 (1.69)	0.527
Invasive mechanical ventilation	5 (3.73)	0 (0)	0 (0)	5 (8.47)	0.037
Noninvasive mechanical ventilation	41 (30.60)	0 (0)	0 (0)	41 (69.49)	0.000

CT: Computed tomography.

The laboratory results of the patients with COVID-19 are summarized in Table 3. An increase in the severity of COVID-19 pneumonia was associated with higher median values of leukocytes (p < 0.001), neutrophils (p < 0.001), D-dimer (p = 0.005), aspartate aminotransferase (p = 0.010), serum urea (p = 0.009), lactate dehydrogenase (p < 0.001), myoglobin (p = 0.026), glucose (p < 0.001), procalcitonin (p = 0.022) and C-reactive protein (p < 0.001), lower platelets count (p = 0.005), lower albumin (p < 0.001) and lower sodium (p = 0.03). The lymphocyte counts were lower in the critical group (p = 0.046). Few patients had co-infections with other viruses, bacteria, etc. The positivity rate of the first test for viral nucleic acid was 33.58%. Furthermore, with an increase in the severity of COVID-19 pneumonia, the positivity rate of the first test for viral nucleic acid increased accordingly (p = 0.010).

**Table 3.** Laboratory test results of patients with COVID-19.

Characteristics	Patients				p-value
	All (n = 134)	Moderate (n = 45)	Severe (n = 30)	Critical (n = 59)	
**Blood biochemical parameters, median (IQR)**					
Leukocytes (3.5–9.5) 10^9^/l	5.42 (3.87–7.43)	4.70 (3.34–6.07)	4.78 (3.78–6.24)	6.94 (4.49–12.54)	0.000
Neutrophils (1.8–6.3) 10^9^/l	3.74 (2.57–6.17)	2.86 (1.83–4.13)	3.54 (2.12–4.63)	5.61 (3.32–10.92)	0.000
Lymphocytes (1.1–3.2) 10^9^/l	1.02 (0.64–1.35)	1.15 (0.91–1.74)	1.16 (0.93–1.34)	0.74 (0.49–1.12)	0.046
Platelets (125–350) 10^9^/l	167.50 (130.75–216.00)	193.50 (160.00–226.00)	165.00 (141.25–228.25)	147.50 (109.75–194.50)	0.005
Hemoglobin (130–175 g/l)	126.00 (116.75–141.00)	126.00 (121.00–139.25)	126.50 (116.75–140.50)	127.00 (110.75–142.25)	0.938
Monocytes (0.1–0.6) 10^9^/l	0.35 (0.25–0.47)	0.36 (0.27–0.44)	0.45 (0.29–0.53)	0.28 (0.21–0.46)	0.734
Activated partial thromboplastin time (20–40) s	29.20 (25.40–32.15)	29.6 (26.05–31.50)	26.40 (24.50–30.20)	30.10 (26.50–34.00)	0.099
Fibrinogen (2–4) g/l	3.06 (2.53–3.69)	2.82 (2.28–3.25)	3.13 (2.52–3.74)	3.34 (2.67–3.91)	0.128
Prothrombin time (9–13) s	11.80 (11.15–12.70)	11.8 (11.15–12.45)	11.40 (10.60–12.10)	12.00 (11.40–13.15)	0.027
International normalized ratio (0.7–1.3)	1.02 (0.96–1.10)	1.02 (0.96–1.08)	0.98 (0.92–1.05)	1.04 (0.99–1.16)	0.021
D-dimer (0–1) μg/ml	0.64 (0.31–1.46)	0.34 (0.17–0.78)	0.57 (0.33–0.88)	1.15 (0.50–5.56)	0.005
Total cholesterol (<5.18) mmol/l	3.58 (3.00–4.50)	3.63 (3.01–4.17)	3.84 (3.14–5.10)	3.40 (2.93–3.94)	0.433
Triglyceride (<1.7) mmol/l	1.06 (0.79–1.54)	1.02 (0.73–1.51)	1.11 (0.78–1.49)	1.11 (0.86–1.73)	0.343
Total protein (65–85) g/l	65.30 (62.70–71.20)	64.80 (63.1–71.2)	64.70 (61.23–71.30)	66.15 (62.15–70.95)	0.900
Albumin (40–55) g/l	38.10 (33.90–41.65)	39.90 (38.30–42.90)	37.00 (34.15–42.55)	34.40 (31.38–39.15)	0.000
Alanine aminotransferase (9–50) U/l	31.10 (13.10–33.20)	21.10 (12.35–34.30)	16.95 (13.03–28.90)	23.70 (15.35–33.75)	0.331
Aspartate aminotransferase (15–40) U/l	25.70 (18.40–36.80)	20.30 (17.48–27.5)	21.55 (16.65–34.00)	31.60 (24.80–45.15)	0.010
Total bilirubin (2–20.4) μmol/l	10.10 (7.10–15.40)	8.35 (6.63–11.60)	9.65 (7.70–15.43)	13.00 (7.75–17.60)	0.182
Serum urea (1.7–8.3) mmol/l	5.07 (3.70–6.71)	4.46 (3.44–5.45)	4.81 (3.47–6.60)	6.13 (4.25–9.46)	0.009
Serum creatinine (57–111) μmol/l	69.05 (56.25–84.23)	63.15 (55.33–79.78)	68.50 (53.85–80.35)	73.10 (58.68–100.58)	0.282
Alkaline phosphatase (40–150) U/l	50.50 (40.75–60.50)	49.00 (41.00–58.00)	50.00 (39.00–56.00)	52.00 (40.75–67.50)	0.570
Sodium ion (137–147) mmol/l	141.05 (137.93–143.28)	142.75 (140.95–143.93)	140.60 (138.70–142.55)	139.90 (136.15–142.38)	0.003
Potassium ion (3.5–5.3) mmol/l	4.14 (3.85–4.46)	4.17 (3.98–4.43)	4.19 (3.72–4.49)	4.12 (3.80–4.49)	0.999
Calcium ion (2.2–2.7) mmol/l	2.19 (2.03–2.35)	2.21 (2.09–2.41)	2.24 (2.05–2.49)	2.13 (2.01–2.27)	0.363
pH value (7.35–7.45)	7.45 (7.42–7.47)	7.43 (7.39–7.47)	7.46 (7.43–7.47)	7.44 (7.42–7.46)	0.265
Partial pressure of carbon dioxide (35–45 mmHg)	35.00 (31.00–39.00)	36.00 (32.00–40.00)	36.00 (31.00–38.00)	34.00 (29.50–38.00)	0.440
Partial pressure of oxygen (80–100 mmHg)	81.00 (58.00–105.00)	100.00 (76.00–109.00)	78.00 (62.00–105.00)	66.00 (51.00–102.00)	0.406
Creatine kinase (38–174) U/l	94.00 (57.00–161.00)	81.00 (47.00–117.50)	83.80 (56.50–151.50)	125.00 (63.50–239.30)	0.064
Lactate dehydrogenase (80–285) U/l	212.00 (164.00–317.00)	168.00 (140.00–217.50)	191.00 (163.00–236.00)	296.00 (212.00–467.50)	0.000
Angiotensin-converting enzyme (12–68) U/l	21.65 (17.03–24.88)	22.70 (17.20–25.58)	20.50 (16.50–23.00)	21.00 (17.05–24.35)	0.645
Myoglobin (14.3–105.5) ng/ml	36.30 (18.05–84.40)	17.55 (12.83–35.28)	36.30 (17.90–91.45)	56.85 (32.85–174.10)	0.026
Troponin (<0.03) μg/l	0.01 (0.00–0.03)	0.003 (0.002–0.015)	0.006 (0.002–0.030)	0.02 (0.009–0.066)	0.058
Glucose (3.9–6.1) mmol/l	5.90 (4.95–8.38)	4.95 (4.36–6.39)	5.48 (5.06–7.86)	7.41 (5.78–11.03)	0.000
Procalcitonin (<0.04) ng/ml	0.09 (0.05–0.25)	0.05 (0.04–0.08)	0.06 (0.05–0.11)	0.22 (0.10–0.52)	0.022
Interleukin 6 (<7) pg/ml	25.52 (9.64–57.32)	7.70 (4.45–10.95)	25.88 (2.22–31.82)	26.88 (9.68–78.67)	0.633
Erythrocyte sedimentation rate (0–15) mm/h	25.00 (15.25–52.00)	25.00 (12.00–40.50)	27.00 (16.00–62.50)	27.00 (17.00–53.00)	0.291
Serum ferritin (21.8–274.7) ng/ml	297.98 (190.98–876.80)	210.20 (80.70–339.70)	244.20 (190.99–876.80)	818.92 (297.98–1339.86)	0.483
C-reactive protein (0–0.5) mg/dl	2.69 (0.94–6.25)	0.98 (0.35–3.28)	2.47 (0.61–4.59)	4.86 (2.36–10.60)	0.000
**Co-infection (negative), (%)**					
Other respiratory viruses	2 (1.49)	2 (4.44)	0 (0)	0 (0)	0.134
Bacteria	8 (5.97)	4 (8.88)	1 (3.33)	3 (5.08)	0.566
Fungus	8 (5.97)	2 (4.44)	0 (0)	6 (10.17)	0.139
Mycoplasma	4 (2.99)	1 (2.22)	2 (6.66)	1 (1.69)	0.400
Chlamydia	3 (2.24)	1 (2.22)	2 (6.66)	0 (0)	0.133
**Viral nucleic acid (negative), (%)**					
**Test for the first time**					
Negative	89 (66.42)	34 (75.56)	24 (80.00)	31 (52.54)	0.010
Positive	45 (33.58)	11 (24.44)	6 (20.00)	28 (47.46)	

**Figure 1.**
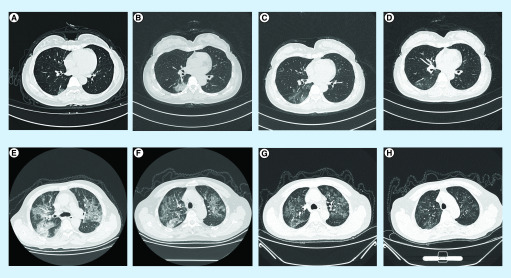
Chest computed tomography images. **(A–D)** A 51-year-old female medical staff. Initially of mild severity, her condition worsened after admission and she finally recovered slowly. **(E–H)** A 66-year-old man. Initially severe, the patient’s condition slowly improved.

A 51-year-old female medical staff tested positive for COVID-19, possibly due to exposure in the hospital and she developed fever and cough that lasted for 3 days. Her chest CT (Figure 1) showed little ground glass opacities below the right lung (Figure 1A). On the 5th day of her admission (Figure 1B), the area of ground glass opacities below the right lung increased. On the 15th (Figure 1C) and 23rd (Figure 1D) days after treatment, the ground glass opacities in the right lung gradually reduced and the lung inflammation slowly resolved. A 66-year-old man infected with COVID-19 due to a meal, presented with fever, cough and shortness of breath for 6 days. His chest CT showed multiple, ground-glass turbidity (Figure 1E). On the 5th (Figure 1F), 9th (Figure 1G) and 20th (Figure 1H) day of admission after active treatment, the ground glass opacities in the lungs gradually reduced and the clinical symptoms improved.

The patients in the critical group were divided into group 1 (dead) and group 2 (surviving). The parameters affecting disease progression (p < 0.05) as in Tables 1– 3 were included in Table 4 to further explore whether disease progression was closely related to the risk of nonsurvivors. The clinical and laboratory indicators, closely related to the severity of patients with COVID-19 are summarized in Table 4. In group 1 and group 2, the median ages were 70.00 and 55.00 years, respectively (p < 0.001). More cases of cardiovascular disease (64.29 vs 35.29%; p = 0.042), ARDS (100.00 vs 52.94%; p < 0.001) and higher median values of D-dimer (1.67 vs 0.49; p = 0.017), lactate dehydrogenase (337.50 vs 258.00; p = 0.026) and glucose (7.85 vs 7.05; p = 0.031), along with lower counts of platelets (median, 134.00 vs 182.00; p = 0.028) and albumin (median, 33.45 vs 39.20; p < 0.001) were found in group 1, as compared with group 2.

**Table 4.** Clinical and laboratory indicators closely related to the severity of patients with COVID-19.

Characteristics	Critical			p-value
	All (n = 59)	Survivors (n = 17)	Nonsurvivors (n = 42)	
**Age, median (IQR), years**	67.00 (56.00–75.00)	55.00 (43.50–61.50)	70.00 (63.75–78.00)	0.000
<60 (%)	19 (32.20)	12 (70.59)	9 (21.43)	0.000
≧60 (%)	40 (67.80)	5 (29.411)	33 (78.57)	
**Chronic disease, (%)**				
Cardiovascular disease	33 (55.93)	6 (35.29)	27 (64.29)	0.042
**Complications, (%)**				
Acute kidney injury	5 (8.48)	0 (0)	5 (11.90)	0.308
Acute respiratory distress syndrome	51 (86.44)	9 (52.94)	42 (100.00)	0.000
**Lung CT images, (%)**				
Unilateral pneumonia	1 (1.69)	0 (0)	1 (2.38)	1.000
Bilateral pneumonia	58 (98.31)	17 (100.00)	41 (97.62)	1.000
**Treatment strategies, (%)**				
Oxygen therapy	59 (100.00)	17 (100.00)	42 (100.00)	1.000
Hormones	48 (81.36)	12 (70.59)	36 (85.71)	0.293
Invasive mechanical ventilation	5 (8.47)	2 (11.76)	3 (7.14)	0.620
Noninvasive mechanical ventilation	41 (69.49)	14 (82.35)	27 (64.29)	0.222
**Blood biochemical parameters, Median (IQR)**				
Leukocytes (3.5–9.5) 10^9^/l	6.94 (4.49–12.54)	4.62 (3.63–11.43)	7.11 (5.38–12.54)	0.298
Neutrophils (1.8–6.3) 10^9^/l	5.61 (3.32–10.92)	3.37 (2.84–8.91)	6.18 (3.61–11.27)	0.240
Lymphocytes (1.1–3.2) 10^9^/l	0.74 (0.49–1.12)	0.95 (0.56–1.16)	0.68 (0.46–1.04)	0.801
Platelets (125–350) 10^9^/l	147.50 (109.75–194.50)	182.00 (119.50–268.50)	134.00 (105.00–186.00)	0.028
Prothrombin time (9–13) s	12.00 (11.38–13.08)	11.80 (11.40–12.25)	12.10 (11.35–13.30)	0.514
International normalized ratio (0.7–1.3)	1.04 (0.98–1.16)	1.03 (0.98–1.07)	1.05 (0.98–1.18)	0.505
D-dimer (0–1) μg/ml	1.06 (0.40–5.54)	0.49 (0.18–0.93)	1.67 (0.66–6.61)	0.017
Albumin (40–55) g/l	34.40 (31.38–39.15)	39.20 (34.25–43.20)	33.45 (30.78–35.85)	0.000
Aspartate aminotransferase (15–40) U/l	31.50 (24.58–45.03)	29.60 (22.55–57.60)	33.40 (25.68–45.28)	0.333
Serum urea (1.7–8.3) mmol/l	6.04 (4.20–9.16)	3.91 (2.90–6.90)	6.55 (4.88–10.39)	0.291
Sodium ion (137–147) mmol/l	139.90 (135.98–142.30)	137.50 (132.90–141.85)	139.90 (136.25–142.95)	0.968
Lactate dehydrogenase (80–285) U/l	291.50 (211.25–458.75)	258.00 (198.00–361.50)	337.50 (213.75–509.50)	0.026
Myoglobin (14.3–105.5) ng/ml	0.00 (0.00–53.88)	0.00 (0.00–27.55)	78.75 (43.25–378.93)	0.051
Glucose (3.9–6.1) mmol/l	7.41 (5.78–11.03)	7.05 (5.15–10.10)	7.85 (6.30–13.10)	0.031
Procalcitonin (<0.04) ng/ml	0.22 (0.10–0.52)	0.17 (0.09–0.36)	0.26 (0.10–0.57)	0.057
C-reactive protein (0–0.5) mg/dl	3.91 (1.52–9.01)	2.21 (1.02–6.28)	6.11 (3.11–11.51)	0.141

Taking nonsurvivors as the dependent variable, the significant risk factors (Table 4) were analyzed using a binary logistic regression. Even after adjusting for age, cardiovascular disease and ARDS, plasma platelets and albumin levels were still associated with the risk of nonsurvivors in the critical group (all p < 0.05; Table 5).

**Table 5.** Risk factors for nonsurvivors in the critical group by binary logistic regression analysis.

Characteristics	Odds ratio	95% CI for odds ratio	p-value	Odds ratio^†^	95% CI for odds ratio^†^	p-value^†^
Age	1.114	1.049–1.183	0.000	/	/	/
Cardiovascular disease	3.300	1.016–10.719	0.047	/	/	/
Acute respiratory distress syndrome	7.539E9	0.000	0.999	/	/	/
Platelets	0.998	0.978–0.997	0.012	0.980	0.963–0.996	0.018
D-dimer	1.112	0.951–1.301	0.185	1.718	0.411–7.183	0.458
Albumin	0.789	0.677–0.920	0.002	0.777	0.614–0.983	0.036
Lactate dehydrogenase	1.004	1.000–1.008	0.073	1.004	0.997–1.011	0.229
Glucose	1.176	0.977–1.417	0.087	1.102	0.855–1.419	0.453

^†^ Adjusted for age, cardiovascular disease and acute respiratory distress syndrome. Patients in the critical group were included in the analysis.

OR: Odds ratio.

**Figure 2.**
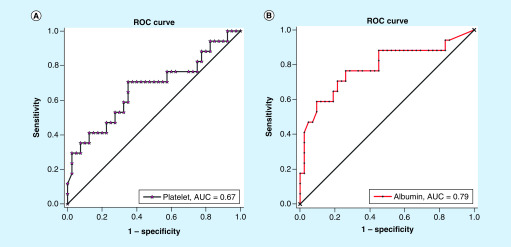
Predictive factors for the risk of nonsurvivors in critical group. The AUC for platelet **(A)** and albumin **(B)** were 0.67 (95% CI: 0.50–0.83; p < 0.05) and 0.79 (95% CI: 0.64–0.93; p < 0.001), respectively. AUC: Area under the curve.

A receiver operating characteristic curve analysis was performed to verify the diagnostic accuracy of the platelets and albumin levels for the risk of nonsurvivors in the critical group. The area under the curve for platelets and albumin were 0.67 (95% CI: 0.50–0.83; p < 0.05) and 0.79 (95% CI: 0.64–0.93; p < 0.001), respectively. The optimal cut-off points for platelet and albumin were 155.00 10^9^/l and 35.1 g/l, respectively. At this level, the Youden index was: 0.36 (platelet), 0.51 (albumin); sensitivity: 70.59% (platelet, 95% CI: 0.44–0.90), 76.47% (albumin, 95% CI: 0.50–0.93); specificity: 65.00% (platelet, 95% CI: 0.48–0.79), 73.81% (albumin, 95% CI: 0.58–0.85). The area under the curve for albumin was higher than that for platelets (Figure 2). Therefore, albumin may be a better predictive marker (cut-off point: 35.1 g/l) for the risk of nonsurvivors in the critical group.

## Discussion

In this study, we identified differences in clinical manifestations, laboratory tests, therapeutic interventions with severity and the risk of nonsurvivors in patients with confirmed COVID-19. We found that albumin may be an independent predictive marker (cut-off point: 35.1 g/l) for the risk of nonsurvivors in critically ill patients with confirmed COVID-19. Our findings highlight the clinical significance of focusing on the levels of albumin as a predictor of a high risk of nonsurvivors in critically ill patients with COVID-19.

Albumin is synthesized by the liver, which plays an important role in maintaining body nutrition and osmotic pressure [13]. A decrease in the levels of albumin is most likely due to liver damage and is likely to be caused by adverse drug reactions and systemic inflammation in critically ill patients with COVID-19. Although, several studies have reported that albumin may predict disease severity in patients with COVID-19 [14,15], we found that in critically ill patients, if the albumin level was less than 35.1 g/l, the risk of nonsurvivors is higher (sensitivity: 76.47%, 95% CI: 0.50–0.93; specificity: 73.81%, 95% CI: 0.58–0.85). However, as hypoproteinemia is defined as blood albumin <25 g/l [16], it will be too late to add albumin at this stage, as the risk of nonsurvivors would be greatly increased. Therefore, we recommend using drugs such as human albumin (that raise levels of albumin) to reduce risk of nonsurvivors, due to decreased levels of albumin (less than 35.1 g/l) in critically ill patients. This may also help clinicians identify patients with high risk of nonsurvivors at an early stage. Low levels of albumin also indicate that the patient’s nutritional status is poor and the body’s immunity is reduced. However, the host’s immune response against RNA viral infection is often weakened due to nutritional deficiencies, which may be overlooked during clinical diagnosis and treatment. Therefore, we recommend verifying the nutritional status of patients with COVID-19 before giving general treatments.

Similar to previous studies [8,10,11], the elderly and male patients with cardiovascular disease had higher morbidity and mortality. Fever and cough were the main symptoms. Elevated levels of neutrophils, D-dimer, lactate dehydrogenase, procalcitonin, C-reactive protein and decreased lymphocyte count were found. However, the elevation of D-dimer was not shown the independent risk factor of a nonsurvivor. In the critical group, there was one case with myocardial infarction, two cases with pulmonary thromboembolism and five cases with ischemic stroke in a group of nonsurvivors, however, there was only one case with ischemic stroke in the group of survivors; after analysis, there was no statistical difference between survivors and nonsurvivors in these three diseases. The proportion of patients without fever with COVID-19 was higher than that found in severe acute respiratory syndrome (SARS) coronavirus (1%) and Middle East respiratory syndrome (MERS) coronavirus infection (2%) [17]. Therefore, if surveillance teams focus on fever detection, patients with COVID-19 may be missed.

The case fatality rate (31.34%) of this study was similar to that by Prof Yu (28.4%), but significantly higher than that from other studies (1.4, 3.2, 11 and 15%) [1,3,8]. In addition, mortality rate in the critically ill was as high as 71.19%, probably because of the difference in sample sizes and case inclusion criteria. This study focused on discharged patients (including rehabilitation discharge and death cases) and no inpatients were included. The patients studied were infected with the first-generation of the virus, but the virus might be more virulent now.

The median hospital stay in the critical group was 1.5 days longer than that in the moderate group. Recently, studies have found that four patients with COVID-19 who met criteria for the discharge requirements had positive real time reverse transcriptase PCR results after 5–13 days in China. This suggests that at a proportion of the recovered patients may still be virus carriers [18]. Therefore, we recommend that recovering patients from the severe and critical groups should have to extend the duration of their hospital stay, so that they can get better rehabilitation services in the hospital.

In this study, the positivity rate for the first test for viral nucleic acid was 33.58%, however, similar to previous studies, the positivity rate increased to 78.36% after multiple tests [19]. These viral nucleic acid test results were inconsistent with the patient’s clinical symptoms and lung CT imaging. This may have been caused by irregular sampling, incorrect PCR amplification and interpretation as well as unstable kits. Therefore, rapidly optimizing the sampling site, ensuring quality of testing kits and standard operating procedure should be done urgently to provide the best infrastructure for the testing of COVID-19.

Previous studies have shown that SARS and Middle East respiratory syndrome coronavirus (MERS-CoV) infection increased the levels of serum proinflammatory cytokines [20,21]. In this study, we noted that higher median values of leukocytes, neutrophils and C-reactive protein were associated with the increased severity of COVID-19 pneumonia, which suggests that a cytokine storm is associated with disease severity. In this study, most patients were prescribed corticosteroids to reduce the inflammatory-induced lung injury. However, previous studies have reported that administering corticosteroids in patients with SARS and MERS did not affect mortality [22,23]. Therefore, we recommend low- and moderate-dose corticosteroids for critically ill COVID-19-positive patients. However, the efficacy and adverse reactions need to be further studied. Although immunoglobulins were used extensively for COVID-19, it should be noted that a third of all critically ill SARS patients developed venous thrombo-embolism including pulmonary embolism during the outbreak in Singapore in 2003 [24], due to an immunoglobulin-induced increase of viscosity in hypercoagulable states. Therefore, immunoglobulins should be used with caution in critically ill patients with significantly elevated D-dimer or decreased platelets to prevent nonsurvivors from pulmonary embolism.

Currently, there are no studies on the beneficial effects of antiviral drugs [10]. Arbidol, oseltamivir, lopinavir and ritonavir treatments are not effective. However, remdesivir is currently in Phase III clinical trials and its efficacy is unknown. At present, some traditional Chinese medicine decoctions were used clinically, as their main action is to improve lung function and enhance host immunity against COVID-19 infection [25].

There were some limitations in this study. First, some cases were incompletely recorded and laboratory testing indicators were not complete, which will cause some deviations. In addition, the sample size may not be large enough and some bias may have occurred. Also, we undoubtedly missed asymptomatic cases, so our study cohort may represent only the more serious COVID-19 cases. Finally, this is a single-center retrospective study, so these results need further confirmation with a multicentric study.

## Conclusion

Albumin levels decreased with the progression of the disease and could be used as an independent predictor (cut-off point: 35.1 g/l) of the risk of nonsurvivors in critically ill patients with COVID-19. This may help clinicians to identify high risk of nonsurvivors among critically ill patients at an early stage.

**Summary points**

**Background**

- Exploring biomarkers closely related to death is of great significance for reducing the mortality of critically ill patients with COVID-19.

**Materials & methods**

- Patients admitted to hospital due to COVID-19 pneumonia were collected and the clinical characteristics and clinical outcomes were analyzed.

**Results**

- The severity of COVID-19 pneumonia was positively correlated with the decrease in levels of platelets and albumin (all p < 0.05). In the critical group, even after adjusting for age, cardiovascular disease and acute respiratory diseases, plasma levels of albumin were still significantly correlated with the risk of nonsurvivors (p < 0.05).

**Conclusion**

- Levels of albumin could be used as an independent predictor of the risk of nonsurvivors in critically illness patients with COVID-19.
